# Tea Polyphenols: A Natural Antioxidant Regulates Gut Flora to Protect the Intestinal Mucosa and Prevent Chronic Diseases

**DOI:** 10.3390/antiox11020253

**Published:** 2022-01-28

**Authors:** Xinzhou Wang, Yanan Liu, Zufang Wu, Peng Zhang, Xin Zhang

**Affiliations:** 1 Department of Food Science and Engineering, Ningbo University, Ningbo 315211, China; 15968052134@163.com (X.W.); liuyanan@nbu.edu.cn (Y.L.); zxdqqzyc@163.com (Z.W.); 2 Department of Student Affairs, Xinyang Normal University, Xinyang 464000, China

**Keywords:** tea polyphenols, antioxidant, intestinal flora, mucosa, chronic diseases

## Abstract

The intestinal tract of a healthy human body hosts many microorganisms that are closely linked to all aspects of people’s lives. The impact of intestinal flora on host health is no longer limited to the gut but can also affect every organ in the body through various pathways. Studies have found that intestinal flora can be altered by external factors, which provides new ideas for treating some diseases. Tea polyphenols (TP), a general term for polyphenols in tea, are widely used as a natural antioxidant in various bioactive foods. In recent years, with the progress of research, there have been many experiments that provide strong evidence for the ability of TP to regulate intestinal flora. However, there are very few studies on the use of TP to modify the composition of intestinal microorganisms to maintain health or treat related diseases, and this area has not received sufficient attention. In this review, we outline the mechanisms by which TP regulates intestinal flora and the essential role in maintaining suitable health. In addition, we highlighted the protective effects of TP on intestinal mucosa by regulating intestinal flora and the preventive and therapeutic effects on certain chronic diseases, which will help further explore measures to prevent related chronic diseases.

## 1. Introduction

According to available research data, the intestinal tract of a healthy adult hosts more than 100 trillion microorganisms, a number equivalent to all the human body cells [1,2]. The complex microbial-host network is established early in life, and it is now indisputable that gut microbes can influence the health of the human gut and even the brain [2,3,4,5]. Therefore, the intestine, which has many microorganisms, is also known as the “second brain” of the human body and plays an irreplaceable role in all aspects of the body’s life activities [6,7]. The studies obtained so far have shown that the composition and structure of the intestinal microorganisms can be influenced by various factors, such as diet, physical activity, age, medication [8,9,10,11]. Among the many factors that affect intestinal flora, dietary factors have received the most attention because of their effectiveness, simplicity, and low cost. Studies have found that different diets will lead to some differences in the intestinal flora and the metabolites of intestinal microorganisms. For instance, the Mediterranean diet (MD), synonymous with a healthy diet, has received strong recommendations from experts because of its healthy nutritional structure [12,13]. Studies have shown that the microbial diversity in the intestinal tract of people on the MD pattern has increased, with an increase in the number of *Bacteroides* and *Lactobacilli* and a decrease in the number of *Firmicutes*; in addition, the MD effectively promotes the production of short-chain fatty acids (SCFAs) by intestinal microorganisms, which is of great help to human health [14,15]. Compared to the healthy MD, the Western-type diet (WD) with higher fat intake also affects intestinal flora and microbial metabolites. By comparing these two dietary patterns, scientists found only a slight difference between the two in terms of intestinal microbial diversity. Still, there is a significant difference between the two in terms of microbial metabolites, which leads to an increase in intestinal permeability in the WD, and this has laid a considerable risk for human health [14,15,16]. The secret of the health benefits of the MD lies in the fact that it is a high residue, low-fat diet, which is more conducive to improving the structure of the intestinal flora and promoting the production of beneficial metabolites [12,15]. Moreover, the nutritional structure of the MD contains a large number of bioactive components, such as polyphenols and polysaccharides [17,18], whose effects on the intestinal flora deserve further study.

As a typical representative of bioactive substances, TP is an excellent natural antioxidant [19,20]. TP is the general name of polyphenolic compounds used in tea; TP has suitable antioxidant, antibacterial, anti-cancer, and other effects, so in various fields have been widely used [20,21]. As research progresses, researchers have discovered a strong relationship between TP and intestinal flora in recent years. Scientists have found that TP has a significant regulatory improvement effect on disordered intestinal flora. The composition structure of intestinal microorganisms has been changed under the influence of TP, through which TP has been widely used in anti-obesity and hypoglycemia [22,23,24]. However, based on the importance of intestinal flora in maintaining health, there should be a broader application for regulating intestinal flora through TP. Therefore, scientists are turning their attention to the protection of the intestinal mucosa. This idea opens a new door for TP to maintain the health of the body by regulating the intestinal flora and also provides new ideas for the prevention and treatment of some chronic diseases [25,26,27].

The intestinal mucosa, part of the immune system, plays an irreplaceable role in maintaining human health. The intestinal mucosa is in constant contact with external antigens and microorganisms, including harmful pathogenic microorganisms. As a critical barrier, the intestinal mucosa keeps pathogenic microorganisms outside and makes specific immune responses to protect the body from damage. In addition, the intestinal mucosa is a necessary pathway for microbial metabolites to enter the body. Therefore, we can visually see that the intestinal mucosa and intestinal microorganisms are closely related [28,29]. With the development of science and technology, our understanding of the intestinal mucosa and the effect of intestinal microorganisms on the intestinal mucosa has improved, and experimental results in recent years have confirmed that the intestinal mucosa and intestinal microorganisms are in an interactive relationship [30,31,32]. Thinking further, we can purposefully modulate the structural composition of microorganisms in the intestinal tract to play a protective role in the intestinal mucosa, strengthening our immunity and providing effective prevention of some related diseases.

Chronic diseases are a general term for diseases that do not constitute an infection and have a long-term accumulation of damage that forms a disease form. Since many people die from chronic diseases every year worldwide, the question of how to treat and prevent chronic diseases more effectively and conveniently is one that we must address [33]. Common chronic diseases include cardiovascular, metabolic, and neurodegenerative diseases; typical representatives include colorectal cancer (CRC), hyperlipidemia, diabetes, Alzheimer’s disease, and inflammatory bowel disease (IBD). Past studies have confirmed a strong link between intestinal flora and various chronic diseases [34]. Given the ability of TP to regulate intestinal flora and the importance of intestinal microbes to the intestinal mucosa, scientists have begun to investigate the prevention and treatment of related chronic diseases by regulating intestinal flora [25,35,36]. To date, there has been some progress in this area of research, which provides a whole new scenario for the prevention and treatment of chronic diseases. In the future, through the regulation of daily dietary composition, the purposeful and appropriate addition of TP and other bioactive substances to regulate the intestinal flora and thus prevent and treat related chronic diseases will gradually become a reality.

The regulatory effect of TP on intestinal flora is still under further research. A variety of new functions are being discovered in the exploration, which can undoubtedly benefit the whole human society. In this review, we outline the mechanisms by which TP regulates intestinal flora and the vital role that intestinal flora plays in maintaining suitable health. In addition, we highlight the role of TP in protecting the intestinal mucosa, treating and preventing some chronic diseases by modulating the structural composition of microorganisms in the gut and provide an outlook on future developments in this field.

## 2. The Relationship between Natural Antioxidant TP and Intestinal Flora

### 2.1. TP

Tea as a beverage in people’s daily lives has a long history. There are many kinds of tea; the most common varieties are green tea, black tea, and oolong tea. The world has a huge consumption of tea every year. Nowadays, tea has become an inseparable part of people’s lives [19,20]. According to modern research, drinking tea is suitable for quenching thirst and very suitable for human health. The beneficial effects of tea on the human body are due to the high content of bioactive substances in tea, including TP, tea polysaccharides, theanine [37,38,39,40], of which TP is the most typical [22,39]. TP is composed of various phenolic compounds in tea leaves together, mainly including flavanols, flavanones, phenolic acids, anthocyanins, etc. Catechins, a group of compounds, are the most important and occupy a large proportion of the TP [41]. Catechins include (−)-epigallocatechin (EGC), (−)-epicatechin-3-gallate (ECG), (−)-epigallocatechin-3-gallate (EGCG) and (−)-epicatechin (EC) [40,41]. As a natural antioxidant, TP has powerful antioxidant and antibacterial abilities and can provide beneficial effects such as weight loss and blood sugar reduction [24,42,43]. Nowadays, TP has been widely used in the production of functional foods, edible films, and the treatment of related diseases [24,43,44]. TP as a research object still contains excellent potential; there are many mysteries that we have not discovered; in recent years, researchers have found that TP has a huge impact on the intestinal flora, based on the importance of intestinal flora for the living body, which opened a new door for the study of TP.

### 2.2. Biotransformation of TP by Intestinal Flora

In our daily life, we can take in TP from outside by drinking tea. The results of in vitro experiments simulating gastrointestinal digestion of TP show that the direct use of TP by the human body is very low, the absorption rate in the small intestine is only 10–20%, and in addition, the investigation also found that after gastrointestinal ingestion of TP, the antioxidant activity measured in the duodenal stage was significantly lower than that in the colonic stage, so it can be inferred that intestinal microorganisms play an extremely critical role in the metabolism and transformation of TP [45,46]. Past studies have confirmed that microorganisms will use a more significant portion of TP in the intestine for further metabolism, and these metabolites are partly absorbed into the blood and partly excreted in the feces [47]. Moreover, studies have shown that intestinal microorganisms can biotransform TP left in the colon, which is further transformed by decarboxylation, demethylation, and dehydroxylation by intestinal microorganisms, eventually producing smaller metabolites, such as phenolic acids [45,48,49]. The flavanols catechins (flavan-3-ols), which is the most important of the TP, undergoes C-ring fission and several dehydrogenations by the action of intestinal microorganisms, resulting in phenylpentanoic acid and phenyl-γ-valerolactones, which is then converted by intestinal microorganisms into various phenols and hydroxybenzoic acids [26,50,51]. Moreover, a study on the in vitro fermentation of EGCG by intestinal microorganisms showed that EGCG underwent sequential ester hydrolysis by intestinal microorganisms and was eventually degraded into a series of metabolites such as 3-(3′,4′-dihydroxyphenyl) propionic acid and 4-phenylbutyric acid [52]. These small metabolites can be absorbed by the intestinal mucosa into the portal circulation, flow to the liver, and then transfer to various body organs [53]. TP has excellent antioxidant, antibacterial and antiviral properties after intestinal microorganisms’ transformation and plays an essential role in maintaining suitable health.

### 2.3. Effects of TP on Intestinal Flora

As an antioxidant, TP has a particular antibacterial effect, which has a certain role in shaping the structure of the composition of microorganisms in the intestine and can affect the metabolites of intestinal microorganisms in the intestine. After research on animal models, human experiments, and in vitro fermentation, the experimental results show that TP can provide some stimulation to some beneficial bacteria in the intestine and inhibit the growth of harmful microorganisms, improving the composition structure of intestinal microorganisms. In addition, TP can also affect the type and content of microbial metabolites in the intestine, promoting the production of beneficial metabolites and reducing harmful metabolites, thereby further maintaining the health of the body [52,54,55,56,57].

So far, many experiments have demonstrated the effect of TP on the regulation of intestinal flora. Liu et al. [52] found that EGCG treatment stimulated beneficial bacteria such as *Christensenellaceae*, *Bifidobacterium*, and *Bacteroides* and inhibited pathogenic bacteria such as *Bilophila*, *Enterobacteriaceae*, *Fusobacterium varium* compared to the blank control group by in vitro fermentation experiments. In another experiment, Yuan et al. [54] provided strong evidence through human experiments that tea consumption can regulate human intestinal flora. According to studies in recent years, it was found that intestinal flora disorders accompany poor diet or inappropriate uses of drugs, and researchers found that this phenomenon can be better improved by using TP. The experiment by Li et al. [23] caused intestinal flora disorders in mice through the use of antibiotics, followed by oral administration of TP to observe the effect of TP on the regulation of disordered intestinal flora. The results showed that TP significantly alleviated the antibiotic-induced decrease in intestinal flora abundance and diversity and increased the relative abundance of probiotic bacteria such as *Eubacterium*, *Roseburia*, and *Lactobacillus*. In addition, TP can also be used to regulate intestinal flora disorders caused by high-fat diet. Wang et al. [58] found that mice fed a high-fat diet showed significant intestinal flora disorders, a decrease in the diversity of mouse intestinal microorganisms, and a significant increase in the *Bacteroidetes* to *Firmicutes* ratio, but subsequent modulation by TP significantly improved the abundance and diversity of microorganisms in the mouse intestine, and also reversed the high ratio of *Bacteroidetes* and *Firmicutes* caused by the high-fat diet. In addition, the experiment also revealed that after the conditioning with TP, the mice showed a significant increase in the levels of butyric acid and acetic acid, both of which are important microbial metabolites. In another experiment, similar experimental results were obtained. The results of Li et al. [22] showed that TP reduced the relative abundance of *Clostridiales* and *Synechococcus* phylum and increased the relative abundance of thick-walled bacteria in the intestine of mice on a high-fat diet. So far, it is indisputable that TP can regulate intestinal flora. From the results, it is clear that this regulation is usually beneficial to human health.

TP has a regulatory effect on intestinal flora and influences the production of intestinal microbial metabolites, including SCFAs, lipopolysaccharides (LPS), and secondary bile acids. Through extensive animal experiments, the researchers found that the content of SCFAs in the intestine of animals treated with TP would be increased, and this phenomenon led to some new thoughts [52,59]. Ding et al. [60] found that six-brewed tea extract increased the abundance of several microorganisms that may produce SCFAs in the intestine of mice, including *Lactobacillus*, *Bacteroides*, and *Ruminococcaceae*, and this finding could provide a solid basis for the increased content of SCFAs in the intestine of animals treated with TP. Moreover, in another study, the researchers obtained the aqueous extract of black tea by steeping it in hot water, and the phenolic compounds were determined by high-performance liquid chromatography to test the inhibition of α-glucosidase and α-amylase activities. It was found that the higher the phenolic compounds, the better the inhibition effect on α-glucosidase and α-amylase activities. Therefore, it was concluded that TP had an inhibitory effect on α-glucosidase and α-amylase activities [61]. We can further consider that most of the SCFAs come from indigestible carbohydrates that reach the intestine through fermentation by intestinal microorganisms, and this experimental result represents that TP can allow more carbohydrates available to intestinal microorganisms to enter the intestine as substrates for the production of SCFAs. SCFAs not only strengthen the body’s immune system but are also chemical mediators of intestinal communication with the brain and play a key role in maintaining the body’s health [62,63]. Secondary bile acids, another important intestinal microbial metabolite, are obtained from primary bile acids that flow into the intestine through a series of transformations by intestinal microorganisms. Experiments by Sinha et al. [64] showed that disturbed intestinal flora inhibited the production of secondary bile acids and contributed to the development of intestinal inflammation. The ameliorative effect of TP on intestinal flora has been demonstrated by experimental results showing that TP increases the abundance of secondary bile acid-producing microorganisms in the intestine, such as *Bacteroides* and *Bifidobacterium* [52]. Secondary bile acids act on immune cells and enhance the body’s immune system [65]. In addition to promoting the production of certain microbial metabolites, TP can also inhibit the production of certain metabolites, such as LPS. Studies have shown that excessive LPS can cause liver damage and related inflammatory responses, while TP can reduce the relative abundance of lipopolysaccharide-producing microorganisms, which can effectively reduce the accumulation of LPS and maintain human health [66,67]. The metabolites of intestinal flora have great potential for further research, and we can hypothesize whether, in the future, we can have beneficial effects on the human body by promoting or inhibiting the production of one or more metabolites? Further research is needed on this point.

**Figure 1:**
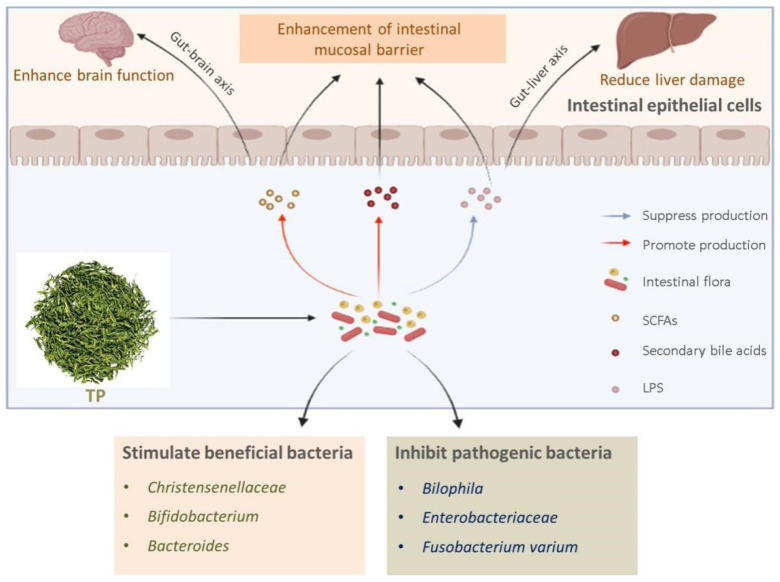
Effect of TP on intestinal flora.

In summary, the interaction between TP and intestinal microorganisms is shown in Figure 1. Interestingly, as research continues to develop, new findings have been made regarding the effects of TP on intestinal flora. Zhou et al. [68] found that TP was able to alter the microbial tricarboxylic acid (TCA) cycle and urea cycle in the rat intestine, and by doing so, improved the energy conversion efficiency of the rats, which was helpful in lowering blood glucose and lowering cholesterol levels. Research on the interaction between TP and intestinal flora continues to advance, and we look forward to another breakthrough in this area in the future.

## 3. TP Regulates Intestinal Flora for the Protection of Intestinal Mucosa

### 3.1. The Importance of Maintaining a Healthy Intestinal Mucosa

As early as decades ago, scientists began to study the association between the intestinal mucosa and human health, and up to now, scientists have concluded that the intestinal mucosa is an important immune barrier in the human body and plays an irreplaceable role in maintaining human health [69,70,71]. The intestinal mucosal barrier consists of four main components, the biological barrier (mainly composed of beneficial microorganisms lodged in the intestine) [72,73], the mechanical barrier (mucus and intestinal epithelial cells (IECs), etc.) [74,75], the chemical barrier (Lysozyme, antimicrobial peptide, secretory phospholipase A2, etc.) [76] and the immune barrier (macrophages, secretory immunoglobulin A, etc.) [77,78]. Let us first look at the mechanical barriers in the intestinal barrier. The mechanical barrier consists of mucus, IECs, and cell junctions. The mucus layer in the intestine is composed of Mucin2 (MUC2) proteins secreted by goblet cells, which form a huge network structure in the intestine [79]. It was found that two layers of mucus usually cover the intestinal epithelium, the outer layer is sparse, and the inner layer is compact [80]. Through their study, Hansson et al. [79] found that the inner mucus layer is free of bacteria, which is good evidence that the mucus layer has a strong ability to block bacteria. IECs and cell junctions form the last line of defense of the mechanical barrier, which prevents pathogenic microorganisms and toxins from reaching here from entering the body and plays the role of absorbing water and nutrients [81]. Antimicrobial peptides, lysozyme, and other antimicrobial chemicals in the intestine together form a chemical barrier, and they are mainly found in the mucus layer, where they play a key antibacterial role [82]. Larsen et al. [83] showed that lysozyme-treated mice reduced the colitis response induced by dextran sulfate sodium (DSS) and effectively maintained intestinal microbial homeostasis compared to normal-fed control mice. In addition, when external harmful substances break through the mechanical and chemical barriers and cause infection, the IECs can send signals to the intrinsic immune cells in the intestinal mucosa, prompting the intestinal mucosa’s immune barrier to function remove the invading harmful substances [84]. The intestinal epithelium and underlying lamina propria have a large number of immune cells, including mast cells, neutrophils, macrophages, T cells, and B cells, which are capable of making an immune response when they receive a signal to remove an antigen by recognizing it and either directly engulfing it or producing secretory immunoglobulin A [85,86]. The biological barrier is mainly composed of beneficial microorganisms lodged in the intestinal tract, which are large in number and can inhibit the growth and reproduction of harmful microorganisms by competing with them for living space with nutrients; in addition, the metabolites of beneficial microorganisms can also play the role of antibacterial and enhance the defense ability of the intestinal mucosa barrier [87,88]. There is much more to the connection between intestinal flora and the intestinal mucosa, as explained in more detail in the next section. In summary, the human intestinal tract maintains a dynamic “offensive and defensive balance,” the importance of maintaining the health of the intestinal mucosa is self-evident; damage to the intestinal mucosa will lead to chronic colitis, colon cancer, and other chronic diseases, seriously endangering our health.

### 3.2. The Relationship between Intestinal Microorganisms and Intestinal Mucosa

It is not surprising that the intestinal flora has a close relationship with the intestinal mucosa, which is in an environment of direct contact with external microorganisms and naturally interacts with the intestinal flora, a view that scientists have long confirmed. Microorganisms in the intestine include beneficial commensal microorganisms and harmful pathogenic microorganisms. An incomplete intestinal mucosal barrier will increase the potential of pathogenic microorganisms to invade the organism. In one experiment, researchers inoculated mice with a defective intestinal mucus layer and wild-type mice with *Citrobacter rodentium*, a laxative pathogenic microorganism. They found that mice with a defective intestinal mucus layer had a higher mortality rate than wild-type mice, suggesting that the lack of a mucus layer leads to an increased chance of infection with pathogenic microorganisms [89]. For that matter, scientists have demonstrated that colonization of the intestinal tract by intestinal flora will affect the formation of the mucus barrier. By way of analogy, the experiments of Wrzosek et al. [90] found that *B. thetaiotaomicron* and *F. prausnitzii* in the gut can regulate the intestinal mucus layer by promoting the differentiation of goblet cell and the glycosylation of mucins, the production and main components of which we have already mentioned in the previous section. The interaction between the intestinal flora and the intestinal mucosal barrier is also reflected in the influence of intestinal microorganisms on the intestinal mucosal immune system. Past studies have found that intestinal flora can activate Toll-like receptors (TLRs), an important class of protein molecules involved in innate immunity that recognize microbes and activate the body to produce an immune response, playing an important role in maintaining gut homeostasis [91,92]. Activation of TLRs triggers the myeloid differentiation primary response protein MYD88 and further leads to the activation of the transcription factor nuclear factor-κB (NF-κB), an important nuclear transcription factor in cells that controls the expression of regulatory genes such as inflammation, immunity, and cell proliferation [93,94]. Danne et al. [95] found that *Helicobacter hepaticus*, a commensal microorganism in the mouse intestine, induces early IL-10 production by macrophages in the intestine and interacts with the receptor TLR2 to exert some anti-inflammatory effects. In addition, the metabolites of intestinal microorganisms have a significant impact on the intestinal mucosal barrier.

By using non-absorbable but fermentable dietary fiber in the intestine, intestinal microorganisms further metabolize it to produce metabolites of SCFAs, commonly including butyric acid, acetic acid, and propionic acid [96,97]. SCFAs play an essential role in maintaining the normal function of the intestinal mucosa. Most intuitively, SCFAs provide energy and maintain the homeostasis of IECs [98,99]. In contrast, the role played by SCFAs in the intestinal immune system has attracted more attention. In the intestine, SCFAs play a role in regulating intestinal mucosal immunity mainly by stimulating G protein-coupled receptors (GPR) on IECs and T cells [100]. Kim et al. [101] performed an experiment in which mice deficient in GPR41, GPR43 and control mice were induced to develop an inflammatory response and fed SCFAs. The experiment results revealed that SCFAs activated GPR41 and GPR43 on IECs and, in turn, activated the value-added protein kinase signaling pathway, which induced the production of chemokines and cytokines during the immune response and helped mice cope with inflammation. Furthermore, studies on butyric acid found that butyric acid can promote regulatory T cells by inducing tolerogenic dendritic cells (DCs) [102]. Thus, SCFAs, as important intestinal microbial metabolites, also maintain a close relationship with the intestinal mucosa.

**Figure 2:**
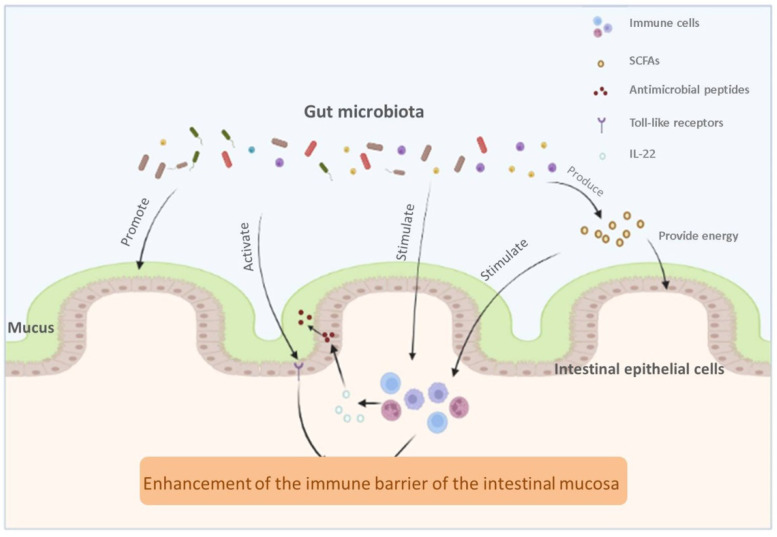
The relationship between intestinal microbiota and intestinal mucosa.

Symbiotic microorganisms in the gut serve as an important biological barrier in the intestinal mucosal barrier, and the relationship between intestinal microorganisms and intestinal mucosa is shown in Figure 2. Of course, although we have made many breakthroughs in this field, there are still many uncharted areas waiting to be explored. We need to think further about what are the hidden associations between intestinal flora and intestinal mucosa that we have not noticed, such as whether intestinal microbes can interact with immune cells that we have not noticed or whether we can use a bioactive substance to purposefully modulate intestinal flora and strengthen the defenses of the intestinal mucosa.

### 3.3. Benefits of TP on Intestinal Mucosa by Intestinal Microbial Structure

The importance of the intestinal mucosa has been known for a long time, and scientists have started long research to discover ways to improve it. In their exploration, scientists found a close relationship between intestinal flora and intestinal mucosa and began to improve intestinal mucosa by regulating intestinal flora. Researchers found that among the various substances that can regulate intestinal flora, TP has numerous advantages, which have attracted the widespread attention of scientists. There is a follow-up further research.

Past studies have pointed out that TP themselves are helpful for the protection of intestinal mucosa; TP can inhibit the generated lipid peroxide radicals, generate less active polyphenol radicals, and terminate the oxidation chain reaction of free radicals. In addition, TP can also improve superoxide dismutase, glutathione peroxidase, and many other antioxidant enzymes, more efficient in removing free radicals [103,104]. The experiments of Grzybowska-Chlebowczyk et al. [105] pointed out that the presence of free radicals is an important factor in triggering IBD. Another investigation showed that Java tea extract effectively scavenged free radicals in mice induced by a high-fat diet and played a protective role against oxidative damage in the intestine of mice [106], so it can be inferred that the effect of TP in removing free radicals can protect the intestinal mucosa from oxidative damage and effectively prevent the occurrence of intestinal inflammation. Nowadays, scientists have turned their attention to intestinal microorganisms, which are closely related to the intestinal mucosa, expecting that the regulatory effect of TP on the intestinal microorganisms can produce some protection for the intestinal mucosa.

Through scientists’ ongoing efforts, new discoveries have been made on the role of TP in protecting the intestinal mucosa by affecting intestinal microorganisms. Firstly, TP has a certain inhibitory effect on harmful microorganisms in the intestinal tract, it was pointed out that TP could inhibit the growth and toxic properties of *Fusobacterium nucleatum* in the intestine, which has been shown to be associated with the development of IBD, and the experimental results showed that TP could prevent the formation of *Fusobacterium nucleatum* biofilm and exert some inhibitory effect on the activity of the biofilm already formed, in addition, the study also indicated that TP was able to attenuate *Fusobacterium nucleatum*-mediated hemolysis and hydrogen sulfide production [107]. Secondly, TP can improve the disorder of intestinal flora and intestinal damage caused by pathogenic microorganisms. Zhang et al. [108] investigated the effects of TP on the regulation of intestinal flora disorders in *Salmonella typhimurium*-infected mice and the mechanism of reducing the damage to the intestinal tract. The experimental results showed that TP reduced inflammation and oxidative stress markers and increased the levels of antioxidant enzymes and tight junction proteins in mice, which effectively improved intestinal flora disorders and reduced the damage to intestinal mucosa. Finally, TP can promote the production of metabolites SCFAs by intestinal microorganisms, thus achieving protection of the intestinal mucosal barrier. Wu et al. [25] regulated the intestinal flora of mice by giving them oral EGCG, and the experimental results showed that the regulation by EGCG significantly increased the relative abundance of SCFAs-producing bacteria (such as *Ackermania*) and the production of SCFAs in the intestine of mice.

In summary, the effect of TP on intestinal microorganisms to protect the intestinal mucosa has been supported by numerous experimental results. In the future, based on the powerful antioxidant ability of TP and their effect on the regulation of intestinal flora, there will be highly effective products with TP as the primary raw material for the prevention or treatment of chronic colitis, colon cancer, and other chronic diseases closely related to the intestinal mucosa. In recent years, the research on the prevention and treatment of such chronic diseases with TP has made new progress and gradually become another new treatment option for patients with related diseases, which is undoubtedly a blessing for patients.

## 4. Intestinal Flora Protects the Intestinal Mucosa to Prevent Related Chronic Diseases

### 4.1. Prevention of Chronic Colitis

IBD is a common chronic inflammatory disease of the intestinal tract that includes mainly ulcerative colitis (UC) and Crohn’s disease (CD) [109,110]. It has been reported that IBD is present worldwide, affecting millions of people worldwide, and is on the rise in countries with westernized lifestyles [111,112]. Even today, the causes of IBD are still unclear. Scientists generally believe that it is caused by multiple factors, mainly environmental, genetic, infectious, and immune factors. In recent studies, it was found that intestinal flora disorders are considered a new factor in the pathogenesis of IBD, which opens a breakthrough in the prevention and treatment of IBD [113]. Nowadays, IBD puts pressure on the health care system of every country, and the prevalence is increasing year by year, so how to effectively treat and prevent IBD has become a major problem that we urgently need to solve.

After a long period of research and exploration, it is known that abnormalities in the immune system of the intestinal mucosa are an important cause of the development of IBD. Given the protective impact of commensal microorganisms in the intestine on the intestinal mucosa and the destructive effect of harmful microorganisms, the treatment, and prevention of IBD by regulating the intestinal flora does have great research potential, and a large number of scientists are now researching this area. Stool samples are often used as a proxy for the microbial composition of the intestine, and studies have found some differences in the microbial communities of stool samples from healthy individuals compared to those from patients with IBD, as evidenced by significantly lower microbial diversity and differences in the composition and structure of microorganisms [114,115,116]. In an experiment that provides strong evidence that a therapeutic effect can be achieved in chronic colitis by regulating intestinal flora, Burrello et al. [117] investigated the effect of fecal microbiota transplantation (FMT) effects on immune-mediated immunity mucosal inflammatory pathways in chronic colitis. The experiment was conducted by using DSS to induce the development of colitis in mice (with symptoms similar to IBD), and then by having the mice orally consume mucus from regular biological donors and feces to modulate their intestinal flora; the experimental results showed that therapeutic FMT was able to reduce colonic inflammation by modulating the expression of pro-inflammatory genes, antimicrobial peptides, and mucins in mice suffering from chronic intestinal inflammation. Thus, they provided the conclusion that FMT can control chronic intestinal experimental colitis by inducing synergistic activation of anti-inflammatory immune pathways. In addition, metabolites of intestinal microorganisms, mainly SCFAs and secondary bile acids, have been shown to have a therapeutic and preventive effect on IBD. The protection of the intestinal mucosa by SCFAs and their role in the intestinal immune system make them closely associated with IBD. Studies have shown that SCFAs maintain intestinal epithelial homeostasis by activating the inflammasome to produce IL-8 and also have an anti-inflammatory effect by modulating immune cells in the intestinal mucosal immune system [118,119,120] and that the production of SCFAs is an important marker of the maturation of the intestinal mucosal immune system [121]. It has been found that the oxidative damage to the intestinal mucosa of patients with UC is caused by insufficient levels of butyric acid, which can be produced by beneficial microorganisms such as Bifidobacterium and Lactobacillus in the intestine [122,123]. Interestingly, Ashton et al. [124] found that by examining the concentrations of SCFAs in stool samples from multiple IBD patients, the concentrations of SCFAs in stool from IBD patients did not show a consistent pattern compared to controls, with some patients showing an increase in both overall and relative concentrations and others showing a decrease in both overall and relative concentrations. The researchers speculate that this phenomenon is due to the varying severity of inflammation in the intestinal tract of the patients; the less severe the inflammation, the intestinal epithelium absorbs, the more SCFAs. This experiment suggests that more factors should be taken into account when investigating the association between SCFAs and IBD. The beneficial metabolites of intestinal flora go far beyond SCFAs; metabolites produced by intestinal flora metabolizing bile acids have also been shown to have some anti-inflammatory effects [125,126]. Thinking further, the wide variety of metabolites of intestinal flora, some of which we have not yet studied, may also have a therapeutic effect on IBD.

Today, drug therapy for IBD is still the primary method; there are some drugs through the regulation of intestinal flora to achieve the anti-inflammatory effect, such as mesalamine [127], but the drugs have certain side effects after long-term use, so scientists have long hoped to find an alternative to anti-inflammatory medications. From the perspective of regulating intestinal flora and protecting intestinal mucosa, TP has received attention. The therapeutic and preventive effects of TP on IBD are mainly reflected in the influence of certain signaling pathways involved in inflammation and the regulation of intestinal microorganisms. The protective effects of TP on the intestinal mucosa and the regulation of intestinal microorganisms have been described in detail above. In addition, as a natural antioxidant, TP has a number of advantages over common anti-inflammatory drugs. First of all, TP can be obtained directly from the diet, which is easy and fast to obtain. In addition, a study comparing TP with sulfasalazine, a drug commonly used to treat IBD patients, showed that TP had fewer side effects than sulfasalazine while playing an anti-inflammatory role [123,128]. It is worth noting that although TP is beneficial to health, it should not be used excessively. For the general population, experts recommend that drinking 10 cups of green tea a day is appropriate [129]. The experiments by Evans et al. [130] suggest that the recurrence of *Clostridium difficile* infection may be associated with tea consumption and that excess TP can also cause a decrease in normal microorganisms in the intestine. Looking to the future, with increased publicity on the benefits of TP, more and more people will adopt the habit of drinking tea in moderation daily, which will effectively reduce the prevalence of IBD.

### 4.2. Prevention of Colorectal Cancer

With the continuous improvement of people’s living standards, the prevalence of CRC is on the rise year by year worldwide. According to relevant reports, colon cancer is the second leading cause of cancer-related deaths worldwide, and how to effectively treat CRC is a problem we have to face [131]. The pathogenesis of CRC is complex and has not been studied so far. Still, there have been several breakthroughs in research on treatment and prevention in recent years.

Past studies have shown that intestinal flora and its metabolites play an important role in protecting the intestinal mucosal barrier and maintaining the balance of the intestinal environment, so it is not surprising to speculate that there is an association between intestinal microbes and the development of CRC. It has been pointed out that the intestinal flora composition of CRC patients is significantly different from that of normal people, as shown by the decrease in beneficial bacteria such as *Faecalibacterium*, *Bifidobacterium*, and the increase in *Mogibacterium*, *Porphyromonas* [132]. This study provides a strong scientific basis that the disturbance of intestinal flora is one of the causes of CRC; therefore, scientists have started to treat and prevent CRC by changing the composition of intestinal flora. Liu et al. [133] performed regulation by adding *Clostridium butyricum* during inflammation induction in mice, and the experimental results showed that the microbial composition of the intestine of mice was changed after regulation by *Clostridium butyricum*, and the abundance of beneficial bacteria increased significantly, and the inflammatory response in the intestine decreased, which effectively prevented the development of colon cancer. In addition, scientists have also found a link between intestinal flora and the innate immune sensor absent in melanoma 2 (AIM2). Studies have demonstrated that AIM2 prevents microbial dysbiosis in the gut and inhibits the uncontrolled proliferation of intestinal stem cells, effectively preventing the development of CRC [134,135]. Man et al. [135] found by 16S rRNA gene sequencing analysis that the composition of intestinal flora was significantly different in wild-type (WT) and *Aim2^−/−^* mice reared alone, with significantly increased levels of *Akkermansia muciniphila* and *Anaeroplasma* in *Aim2^−/−^* mice compared to WT mice, and the levels of *Anaerostipes*, *Bifidobacterium*, *Flexispira*, *Prevotella*, and *Paraprevotellaspecies* were decreased. Moreover, the experiments also used the propagation properties of the intestinal flora. They found that the number of tumors and cancer incidence was significantly lower in the colon of *Aim2^−/−^* mice co-housed with WT mice compared to *Aim2^−/−^* mice housed alone. This experiment provides strong evidence that intestinal flora can interact with AMI2 to prevent CRC development.

Compared with traditional radiation therapy, the treatment and prevention of CRC by regulating intestinal flora have the advantages of being more economical, more convenient, and safer, but the effectiveness needs to be further studied. However, it certainly provides a new possibility for CRC treatment and prevention, and this treatment method is also more acceptable to people, which is of great significance to reduce the incidence of CRC.

## 5. Conclusions

As a natural antioxidant, TP can protect the intestinal mucosa, regulate intestinal flora, and promote the production of beneficial metabolites by intestinal microorganisms. The homeostasis of intestinal flora and the production of beneficial metabolites can effectively protect the intestinal mucosal barrier, enhance the immune defense of the intestinal mucosal barrier, and effectively prevent and treat IBD, CRC, and other related chronic diseases. The therapeutic approach of regulating intestinal flora through the use of TP has been noted to have several advantages over traditional treatments for IBD and CRC, but whether this method can replace the traditional method and achieve the same therapeutic effect requires more in-depth research.

## Data Availability

The data presented in this study are available in review.
